# 

**Published:** 1892-10

**Authors:** 


### *EDITORIAL.*

The office of THE JOURNAL is in rooms 44 and 45 Old Capitol Building.
Address communications relating to the Editorial Department to DR. L. B. GRANDY, Atlanta, Ga., Box 431.
Business communications should be addressed to DR. M. B. HUTCHINS, Atlanta, Ga.
All remittances should be made payable to "ATLANTA MEDICAL AND SURGICAL JOURNAL."
Articles for publication should reach this office not later than the 15th instant.
The editors are not responsible for views expressed by contributors.

### TO OUR SUBSCRIBERS.

As customary heretofore we send bills this month (October) to each subscriber who is due for any time preceding. We send them at this time because most of our subscribers get in their money during the fall and winter.

We are giving you the best medical journal in the country for the subscription price, $2.00 per annum, our present rate. All bills are made out upon the basis of $2.00 per annum. You will find it easier to pay year by year than to let the account run on, the amount doubling each year, until finally you decide it is all right not to pay at all, and have a big blank on the creditor's side of the account.

We beg you to keep your receipts, and notify us of any error so we may correct it in your favor. Please send the bill with the money to be receipted. Those of you who get THE JOURNAL "on time" pay no more than cash subscribers.

To those who pay promptly: We thank you, and shall do our best to keep you with us by giving you a better JOURNAL than ever.

To those somewhat in arrears: We thank you for what you have paid, and believe you mean to and will pay the balance.

To those who have paid nothing: We don't think you meant to get your JOURNAL for nothing, but have "just been putting it off," perhaps to begin settling up this fall.

We will keep THE JOURNAL up and improve it, and will continue to consider the doctors our best friends.

### TRI-STATE MEDICAL SOCIETY.

Following is a list of the papers to be read at the fourth annual meeting of the Tri-State Medical Society, of Alabama, Georgia and Tennessee, which will be held in Chattanooga, October 25, 26, and 27, 1892:

"Eye Symptoms in General Disease," by J. L. Minor, M. D., Memphis, Tenn.

"Talipes Equino-varus" (with presentation of patients), by C. W. Barrier, M. D., Rome, Ga.

"Sequences of Cystitis Media Purulenta," by T. Hilliard Wood, M. D., Nashville, Tenn.

"Report of One Thousand Five Hundred Strabismus Operations, with Some Observations on the Same," by A. W. Calhoun, M. D., Atlanta, Ga.

"Special *vs.* General Practice in Medicine," by W. J. Killen, M. D., Birmingham, Ala.

"Synovitis," by J. B. Cowan, M. D., Tullahoma, Tenn.

"A Plea for Higher Medical Education in the South," by Luther B. Grandy, M. D., Atlanta, Ga.

"Pharmaceutical Preparations of the Present Day," by John C. Le Grand, M. D., Anniston, Ala.

"A Clinical Study of the Relation Between Scarlet Fever and Diphtheria," by W. D. Hoyt, M. D., Rome, Ga.

"The Treatment of Inguinal Hernia," by J. W. Handly, M. D., Nashville, Tenn.

"Fistula in Ano," by Andrew Boyd, M. D., Scottsboro, Alabama.

"Stricture of the Male Urethra; its Diagnosis and Treatment," by W. L. Gahagan, M. D., Chattanooga.

"Phymosis," by Erasmus T. Camp, M. D., Gadsden, Ala.

"The Rational Treatment of Enlarged Prostate in Old Persons," by George W. Broome, St. Louis, Mo.

"Advanced Theories in Psychical Science," by John H. Purdon, M. D., Cullman, Ala.

"Drunkenness, and its Gold Cure," by John P. Stewart, M. D., Attala, Ala.

"Surgery; Things to Do and Things Not to Do," by Willis F. Westmoreland, M. D., Atlanta, Ga.

"A Few Selected Cases in Laparotomy," by W. H. Wathen, M. D., Louisville, Ky.

"Extra-Uterine Pregnancy," by Richard Douglas, M. D., Nashville, Tenn.

"The After-treatment of Abdominal Operations," by W. E. B. Davis, M. D., Birmingham, Ala.

"Hepatic Abcess," by W. B. Ward, M. D., Selma, Ala.

"Report of Treatment of Sterility," by J. M. Head, M. D., Zebulon, Ga.

Titles not announced: John L. Sowell, M. D., Knoxville, Tenn.; J. M. Masters, M. D., Knoxville, Tenn.; C. S. Briggs, M. D. Nashville, Tenn.; I. N. Love, M. D., St. Louis, Mo.

The committee has secured a one-and-one-third rate from the railroads on the certificate plan. The meeting promises to be one of unusual interest.

